# Combustion Characteristics of Low Calorific Value Biogas and Reaction Path of NOx Based on Sensitivity Analysis

**DOI:** 10.3389/fchem.2021.830329

**Published:** 2022-02-16

**Authors:** Zongliang Zuo, Chuanjia Qi, Jinshuang Ma, Huiping Sun, Siyi Luo, Dongdong Ren, Yifan Zhang, Jianxiang Guo, Zhanjun Cheng, Chang Li

**Affiliations:** ^1^ School of Environmental and Municipal Engineering, Qingdao University of Technology, Qingdao, China; ^2^ School of Energy and Power Engineering, Shandong University, Jinan, China; ^3^ School of Environmental Science and Engineering, Tianjin University, Tianjin, China

**Keywords:** biogas combustion, sensitivity analysis, reaction path, NOX, biomass

## Abstract

The combustion mechanism of biogas mixture is unclear, which leads to the lack of basis for the control of operating parameters. Combustion characteristics and reaction path of typical low calorific value biogas with variation of preheating temperature and air equivalence ratio (*Φ*) are discussed in this paper. Preheating can not only improve the flame propagation speed and flame temperature, but also increase the proportion of NO in the product at the end of combustion flame. To some extent, improving combustion efficiency and NOx control are contradictory operating parameters. The amount of NO increases with the increase in flame distance. The maximum value of NO appears when *Φ* is 1.1. NO formation rate is improved by preheating the biogas. The paths of N_2_ → N_2_O →NO, N_2_ → NNH →NO, and N_2_ →NO are all enhanced. When the equivalence ratio changes from 1.0 to 0.8, NO formation rates decrease.

## Introduction

Biomass energy is a renewable energy with zero carbon emission in the ecological sense. It is the only renewable green energy that can be transported and stored efficiently (Gupta et al., 2010). Biomass resources are widely used in various forms, accounting for about 14% of the global total energy consumption, and has become the fourth largest energy after coal, oil, and natural gas (Lim et al., 2012). According to experts’ prediction, biomass energy consumption will account for 38% of the total global fuel consumption by 2050, while the power generation using biomass energy will account for 17% of the total global power generation (Demirbas, 2007; Whistance, 2012).

There are three main sources of NOx in the fuel process: thermal-Nox, fuel-Nox, and fast-NOx. The generation of thermal NOx is significantly affected by temperature factors. Some studies have shown that when the combustion temperature is lower than 1,573 K, the generation of thermal NOx can hardly be observed. Part of the NOx released by fuel combustion comes from the N contained in the fuel. The N content of biomass fuel is lower than that of coal fuel. However, the amount of NOx produced by fuel accounts for about 75%–90% of the NOx produced by fuel combustion (Mao et al., 2019). As the oxidation reactions of volatile nitrogen and coke nitrogen include hundreds of steps, the NOx formation mechanism is complex. Fast-NOx is the CH atom group generated by hydrocarbons in the fuel at high temperature, which collides with N_2_ molecules in the air, resulting in the formation of HCN compounds.

Some studies have reported that about 70%–100% of N in fuel will eventually be converted to NO during combustion (Li and Chyang, 2020). Compared with traditional fossil fuels, the mass fraction of nitrogen in straw biomass fuel is generally high (Stolarski et al., 2013). The N content of straw can reach about 0.7%. Therefore, it is urgent to develop efficient biomass technology and reduce NOx emissions.

At present, the efficient NOx control methods are low NOx combustion technology and flue gas denitration technology. For some small capacity combustion boilers (such as evaporation <5 t/h), it is difficult to use denitration instruments. Under these circumstances, NOx can only be controlled by low NOx combustion technology. Therefore, low nitrogen control method and mechanism research in combustion process is the key to reduce NOx for small-capacity combustion boilers.

At present, there are five kinds of low NOx combustion technologies: gasification combustion, low NOx burner, fuel re-burning, air staged combustion, and flue gas recirculation (Ti, 2015; Li and Chyang, 2020). Gasification combustion is the most effective combustion technology. Air staged combustion technology is the easiest way to achieve. The cooperative control between them will be a promising low nitrogen combustion technology for some small-capacity combustion boilers. However, the transformation mechanism of NOx under different conditions is not clear.

The mechanism of chemical reaction describes the way that fuel and oxidant produce reaction products through a complex reaction process. According to the detailed degree of reaction mechanism describing the chemical reaction process, the reaction mechanism can be divided into three types: general reaction mechanism, detailed reaction mechanism, and simplified reaction mechanism (Stanley et al., 2013). At present, biogas is mainly obtained by pyrolysis or gasification of biomass or other solid wastes (Zuo et al., 2019; Zuo et al., 2021a; Zuo et al., 2021b). Biogas is usually composed of CH_4_, CO, CO_2_, and H_2_ (Pio et al., 2020).

In order to describe the combustion process of biogas, the reaction mechanism must be able to describe the combustion process of CH_4_, CO, CO_2_, and H_2_ respectively (Fischer and Jiang, 2015). The gas research institute mechanism (GRI) is a detailed reaction mechanism that can describe the combustion process of biomass gas accurately. GRI is a detailed mechanism, developed by the University of California Berkeley, Stanford University, the University of Texas at Austin and SRI International^1^. GRI-mech 3.0 contains 53 components and 325 elementary reactions, which can describe the reaction process of C1–C3 and the formation mechanism of NOx and other pollutants. The mechanism has high academic value, accuracy, and credibility.

At present, the formation mechanism research is mainly by simulation calculation (Fischer and Jiang, 2014) (Fischer and Jiang, 2014). The study of combustion mechanism only focuses on the combustion of a single component or CO/H_2_ binary fuel (Fischer and Xi, 2016; Akih-Kumgeh and Bergthorson, 2013) (Fischer and Xi, 2016; Akih-Kumgeh and Bergthorson, 2013) (Fischer and Xi, 2016; Akih-Kumgeh and Bergthorson, 2013) (Fischer and Xi, 2016; Akih-Kumgeh and Bergthorson, 2013) (Fischer and Xi, 2016; Akih-Kumgeh and Bergthorson, 2013). At present, different developers have carried out combustion simulation of CO/H_2_/CH_4_ single-component fuel. Fisher et al. (Fischer and Xi, 2014; Fischer and Xi, 2016) and Lee et al. (2015) evaluated the prediction performance of GRI3.0, DRM22, NUI-NG, USC-Mech-II, SanDiego, and Aramco mech 1.3 for CO/H_2_/CH_4_/CO_2_ mixed fuel, respectively. According to the reaction mechanism of CO/H_2_/CH_4_ mixture, Akih-Kumgeh and Bergthorson (2013) and Nikolaou et al. (2013) simplified the USC-mech-II and GRI 3.0 mechanisms, respectively, by sensitivity analysis to obtain the corresponding skeleton mechanism. However, it is found that there is no unified understanding of the detailed combustion mechanism and performance of biogas. Up to now, some scholars have studied the combustion characteristics of biomass gas with simple components as the model of biomass gas. For example, Gao et al. (2020) studied the combustion characteristics of CH_4_/air by numerical simulation. By changing the burner structure, operating parameters, and physical properties of alumina pellets, the catalytic combustion results were discussed. Furthermore, the addition of inert gas can inhibit the conversion of NOx. Influence of chemical kinetics on predictions of the performance of syngas production from fuel-rich combustion of CO_2_/CH_4_ mixture in a two-layer burner was discussed in Shi’s study (Shi et al., 2019). The combustion characteristics of few simple components are difficult to express the combustion characteristics of complex combustion components. Based on this, the calculation of the laminar flame speed of the gas from lignocellulosic biomass gasification, using two different reaction mechanisms, was studied by Hernández et al. (2004). Varying both the gas composition and the excess air within the engine, the flame speed was calculated by using the Chemkin code and obtaining correlations depending on pressure and temperature.

The combustion mechanism of biogas mixture is unclear, which leads to the lack of basis for the control of operating parameters (Li et al., 2020). Compared with the conventional syngas composed of CO and H_2_, the CH_4_ content in biogas cannot be ignored, and its combustion reaction mechanism is significantly different from that of conventional syngas. The reaction mechanism is obviously different from that of syngas with conventional CO/H_2_ binary fuel. According to the actual composition of biomass produced syngas, it is necessary to develop its combustion reaction mechanism.

Nitrogen oxides are important pollutants in biomass gas combustion, and the studies on combustion characteristics and NO_X_ formation path of syngas flame with complex components are relatively scarce seldom. Different from their research, in order to further improve the representativeness of gas components, real gas components for characteristic calculation and analysis were used in this research. The study attempts to discuss the effect of staged combustion on the conversion of nitrogen oxides in biomass gas. In this paper, the laminar premixed combustion characteristics of biogas are studied; in particular, the biogas that is closer to the real combustion condition is selected for simulation study. The combustion characteristics of biogas under different preheating temperature and air equivalence ratio. The skeleton mechanism of nitrogen oxide conversion under different conditions are obtained, which provides the basis for low nitrogen combustion technology.

## Materials and Methods

### Materials

As the typical pyrolysis gas of straw biogas was selected, its composition was obtained through a pyrolysis experiment with a temperature of 700°C. As shown in Table 1, the biogas was collected and the composition was detected by an infrared gas analyzer (Gasboard-3100P, resolution ratio 0.01%, error ±1% FS).

**TABLE 1:** Gas composition, vol%.

CO	H_2_	CH_4_	N_2_	CO_2_	Calorific
%	%	%	%	%	kcal/Nm^3^
9.35	13.05	7.5	36.5	33.6	1,260

### Methods

#### Combustion Reactor

In this paper, the premix reactor was selected as the flame simulation reactor to study the combustion characteristics of one-dimensional premixed laminar flame (Shen et al., 2010). The main purpose of the reactor is to study the combustion characteristics of quasi one-dimensional, planar, adiabatic laminar premixed flame under constant pressure. The model function of the reactor was used to study the influence of working conditions on combustion temperature and free radical concentration. Moreover, the parameter study function was used to study the influence of parameter changes on flame spreading speed (Nikolaou et al., 2014). Sensitivity analysis of flame temperature and NOx formation was also carried out in the reactor. Finally, the pathway of NO production was analyzed.

In this reactor, the pseudo one-dimensional laminar premixed flame is studied. For each reaction mechanism, the premix reactor is selected to simulate the adiabatic laminar premixed flame, and good simulation results can be obtained.

#### Mechanism Document

Two mechanism files are used in this simulation, one is the mechanism file used in the flame speed parameter study case (FSPSC) of CHEMKIN software, and the other is the mechanism file of GRI-mech 3.0 methane.

The FSPSC mechanism file contains C, H, O, and N elements, and the components include all the components in the biogas, which can simulate the combustion process of the selected biogas. Since the mechanism document does not involve the formation of NOx, it can only be used to study the influence of working conditions on flame combustion temperature and flame spreading velocity.

For the variation of nitric oxide and nitrogen dioxide concentration, the GRI-mech 3.0 methane mechanism file was selected. The file includes 53 components and 325 elementary reactions. The temperature range of the GRI-mech 3.0 methane mechanism file is 1,000–2,500 K, the pressure range is 10 Torr to 10 bar, and the equivalence ratio (*Φ*) range is 0.1–5. GRI-mech 3.0 mechanism file of GRI-mech 3.0 contains the formation reaction of NOx, which was used to study the influence of working conditions on the concentration distribution of nitric oxide and nitrogen dioxide, to analyze the sensitivity of flame temperature and the formation of NOx in this paper.

## Results and Discussion

### Effect of Preheating Temperature on Biogas Combustion Characteristics

Due to the low calorific value of biogas, it is difficult to ignite and the combustion temperature of this biogas is low. Air or fuel preheating is an effective method to improve combustion temperature and thermal efficiency of the furnace. The effects of preheating temperature (Tp) on flame combustion characteristics were studied in this section. Ambient temperature (Ta) = 298 K, pressure (P) = 1 atm, equivalence ratio (*Φ*) = 1, and the fuel inlet velocity (Vf) is 50 cm/s.

**FIGURE 1:**
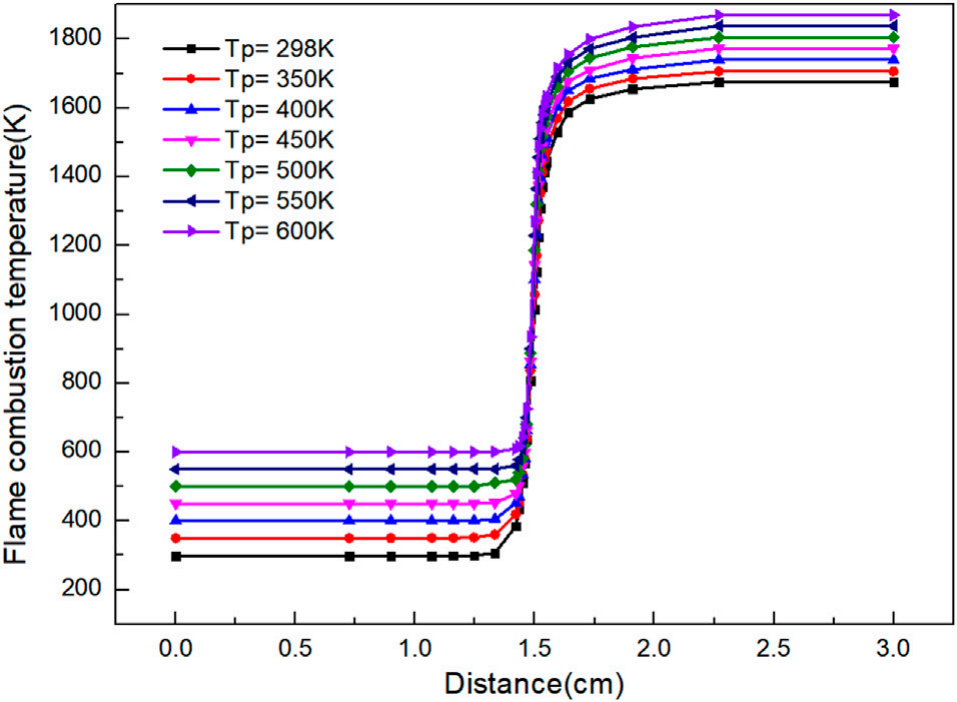
Graph of flame combustion temperature versus flame distance with variation of preheating temperature.

It is clear from Figure 1 that the final flame temperature of laminar premixed flame increases with the increase of gas preheating temperature. When the gas is burned at 298 K without preheating, the minimum temperature of flame is 1,676 K. With the increase of preheating temperature, the final flame temperature moves up as a whole. When the preheating temperature is 650 K, the maximum combustion flame temperature is 1,899 K.

**FIGURE 2:**
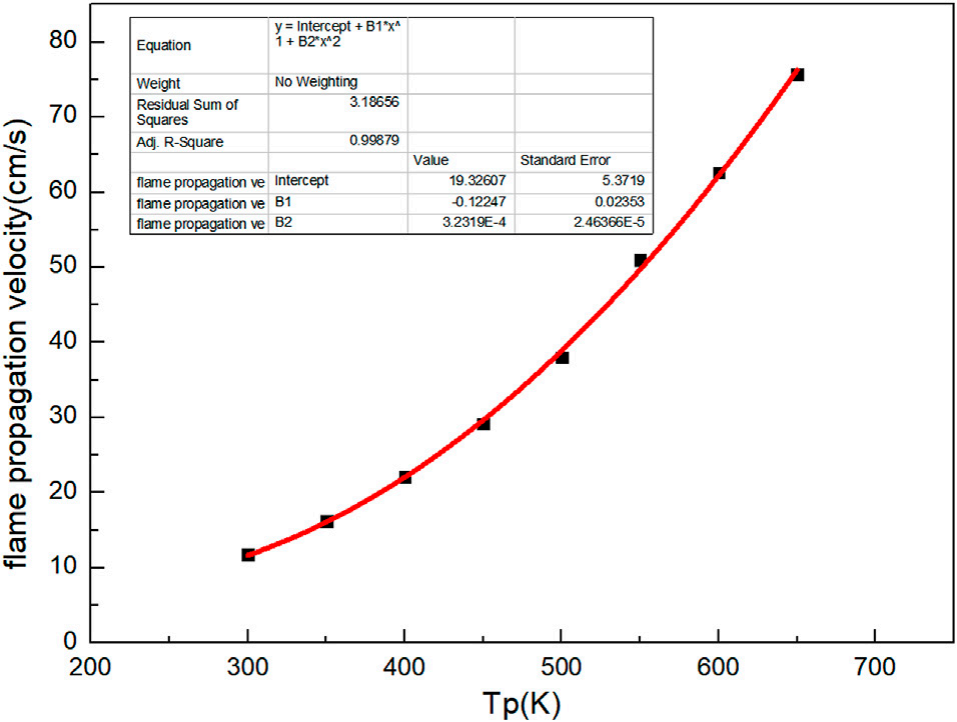
Curve of flame velocity with preheating temperature.

As shown in Figure 2, the preheating temperature of the biogas has a great influence on the flame propagation velocity. The flame propagation velocity increases exponentially with the increase of preheating temperature (Vu et al., 2011). The flame propagation velocity is 11.74 cm/s without preheating. When the preheating temperature is 650 K, flame propagation velocity is 75.7 cm/s, about 5 times that without preheating. There is a quadratic function relationship between the combustion flame propagation velocity and the preheating temperature.

$$\mathrm{Vf}=19.32607-0.12247\times \mathrm{Tp}+3.2319\;\mathrm{E-4}\times Tp^{2}$$ (1)

Where Vf is combustion flame propagation velocity, cm/s; Tp is preheating temperature, K.

**FIGURE 3:**
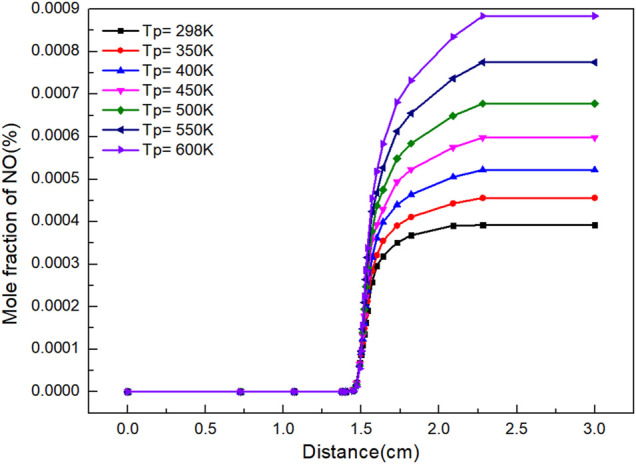
Graph of NO mole fraction with distance under different preheating temperatures.

NO is the typical and most important oxide in NOx. It reflects the reaction of NOx to a certain extent. It can be seen from Figure 3 that the mole fraction of NO increases with the increasing distance and the continuous reaction. When flame distance is 1.4 cm, the amount of NO produced by the reaction increases rapidly. Preheating improves the concentration of NO. This is because preheating increases the flame temperature, thereby improving the generation of thermal NOx. The higher the preheating temperature, the higher the flame temperature at the same position is. The mole quantity of NO and the reaction rate of NO formation reaction are closely related to flame temperature, which is affected by preheating temperature.

**FIGURE 4:**
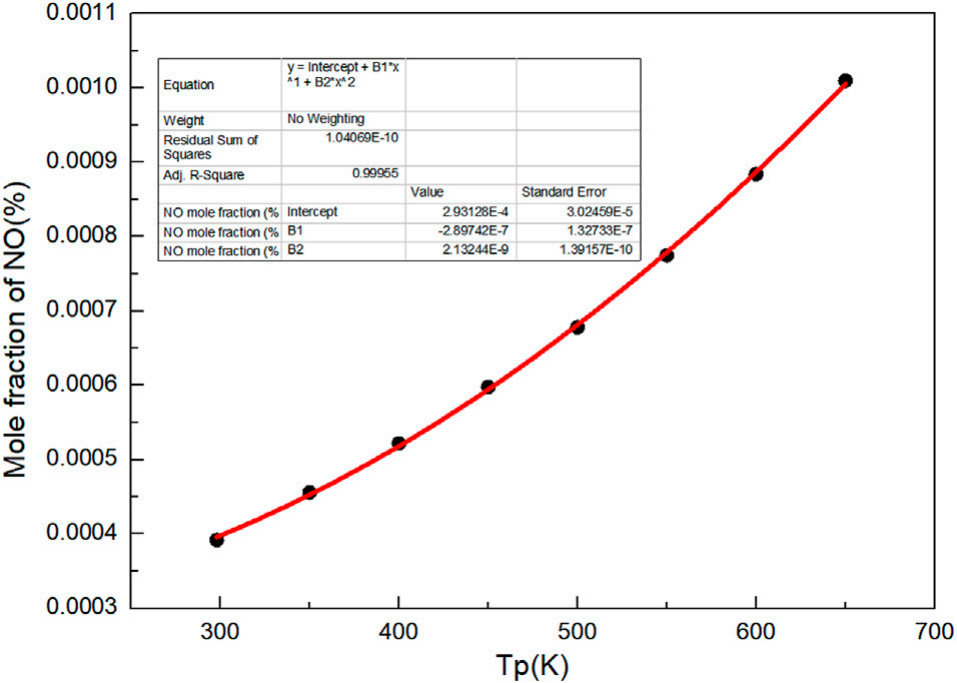
Curve of NO mole fraction with preheating temperatures.

Besides, as shown in Figure 4, with the increase of preheating temperature, the mole fraction of NO increases. It can be seen that increasing the preheating temperature of gas can not only improve the flame propagation speed and flame temperature, but also increase the proportion of NO in the product at the end of combustion flame. There is a quadratic function relationship between the preheating temperature and NO mole fraction.

$$n\left(\mathrm{NO}\right)\%=2.93128\mathrm{E-4+2}\mathrm{.89742}\;\mathrm{E-7}\times \mathrm{Tp}\mathrm{+2}\mathrm{.13244}\;\mathrm{E-9}\times Tp^{2}$$ (2)

Where n (NO)% is the mole percentage of NO, %.

**FIGURE 5:**
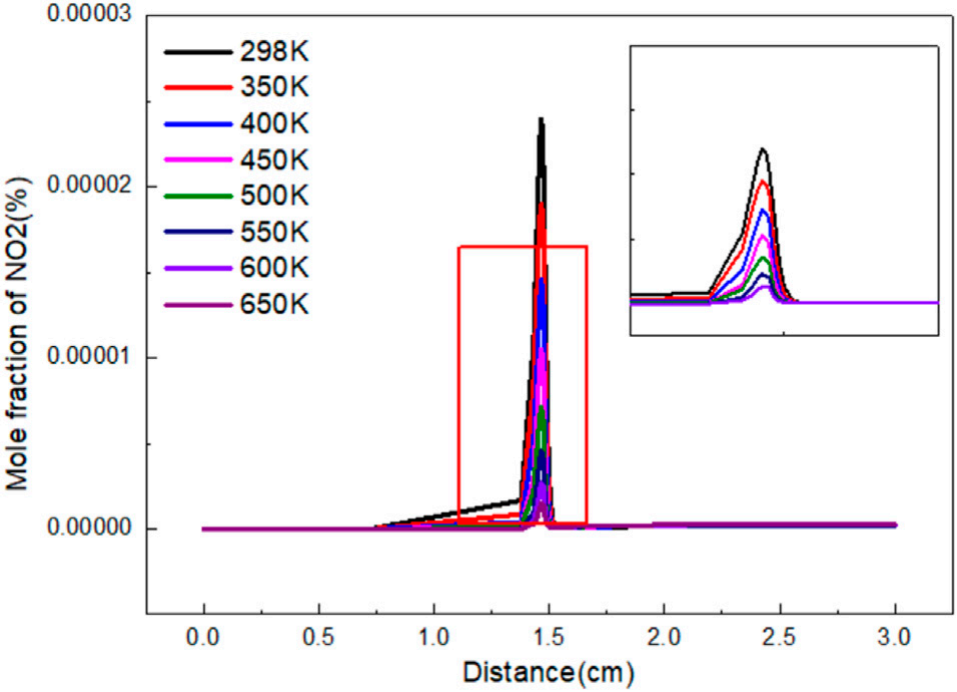
Variation of NO_2_ mole fraction with flame distance under different preheating temperature.

Figure 5 shows the variation curve of NO_2_ mole fraction with distance under different gas preheating temperatures. Under different preheating conditions, the change trends of the mole fraction of NO_2_ are almost the same. Firstly, the mole fraction of NO_2_ increases continuously with the progress of the reaction. After reaching the peak value, NO_2_ reduces and finally reaches a stable value. The final mole fraction of NO_2_ is the highest when there is no preheating temperature. When fuel or air heated, the final mole fraction of NO_2_ decreases sharply at the center of the flame. The final mole fraction of NO_2_ increases with the increase of preheating temperature. However, the effects are weak.

### Effect of Air Equivalence Ratio on Biogas Combustion Characteristics

Staged combustion is an important method of low nitrogen combustion. The adjustment of *Φ* is the realization of staged combustion. The effects of *Φ* on flame combustion characteristics were studied in this section.

**FIGURE 6:**
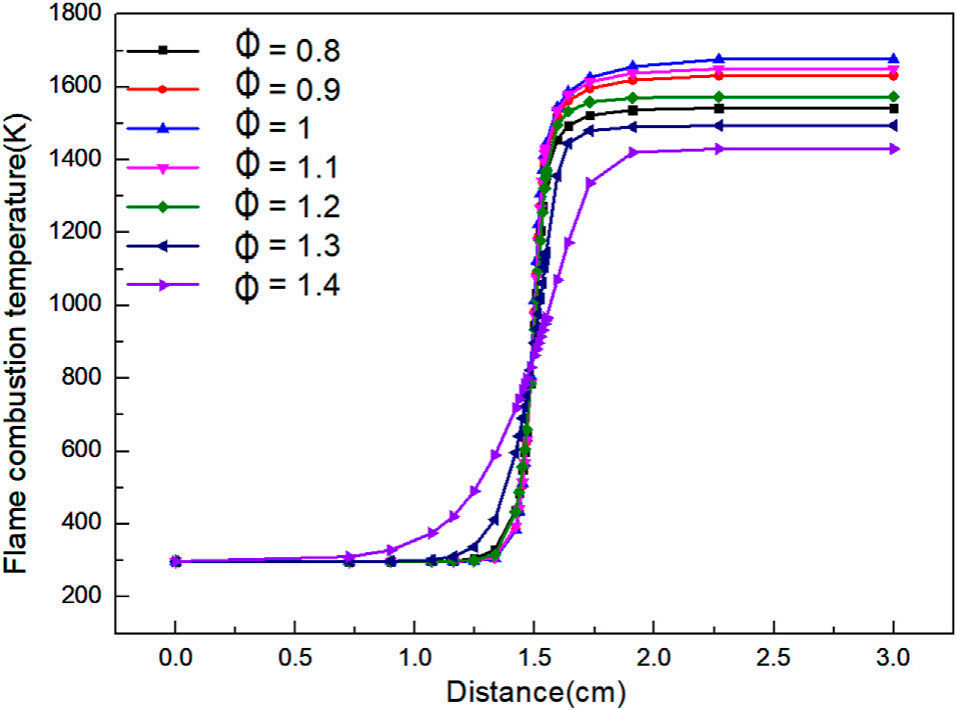
Graph of flame combustion temperature versus flame distance with variation of *Φ*.

As shown in Figure 6, when the flame distance is 0–0.7 cm, the combustion temperature of the flame is basically stable at the initial. When the flame distance is 0.7–1.8 cm, it is the front of the flame, the flame temperature increases rapidly. When the flame distance is 1.8–2.6 cm, the flame temperature rises slowly and gradually reaches chemical equilibrium. When *Φ* is 1.0, the final flame temperature can reach the highest value of 1,675 K, and the final flame temperature is 1,430 K.

When *Φ* < 1, the actual amount of air supplied exceeds the theoretical amount required, which leads to the decrease of fuel concentration. The excess air will absorb heat in the combustion process, which eventually leads to the decrease of flame temperature.

When *Φ* > 1, the actual air supply is lower than the theoretical air demand, which leads to incomplete combustion of biogas due to lack of oxygen, and eventually reduce the flame temperature. With the increase of *Φ*, the temperature of biogas begins to increase in a closer distance, but the heating rate of the flame is slower. Thus, the reaction interval is longer, and the highest temperature of the reaction is lower. It can be concluded from the graph that a very high or a very low *Φ* is not conducive to the combustion reaction, which will reduce the final temperature of the reaction, and the farther *Φ* is away from 1, the more the flame temperature decreases. However, within the temperature range required by the reaction, the control of temperature will play an indirect role in the control of NOx.

**FIGURE 7:**
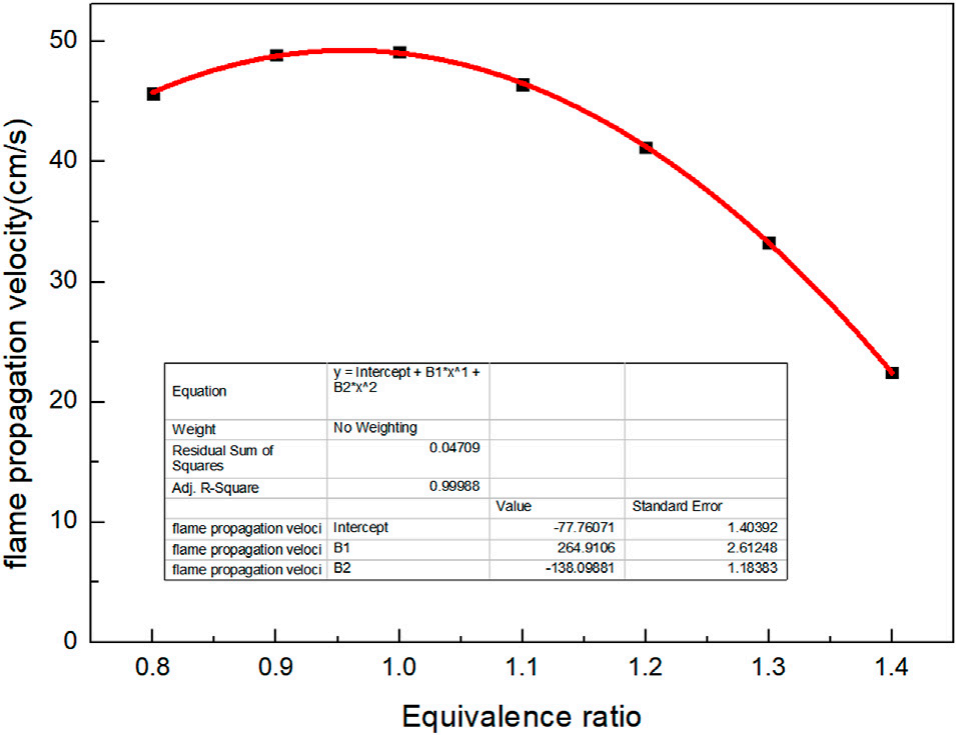
Curve of flame velocity versus *Φ*.

Because the calorific value of the selected biomass gas is low, the biogas cannot be lighted when air supply is changed. To measure flame velocity, the typical low calorific value biogas was preheated at 550 K, and the curve of flame combustion velocity with *Φ* is obtained, as shown in Figure 7. It can be seen from the figure that the overall trend of flame propagation velocity increases firstly and then decreases with the increase of *Φ*. Due to insufficient air, the premixed gas does not have complete combustion when *Φ* < 1. Due to excessive air, the fuel cannot be diluted sufficiently when *Φ* > 1. The minimum propagation velocity of flame is 22.5 cm/s when *Φ* is 1.4. The combustion propagation velocity is related to the problem of backfire and the ignition of burner, so the influence of *Φ* on flame propagation velocity must be paid attention to in combustion. At the same time, when using CHEMKIN software to simulate, it is found that the *Φ* of biogas with low calorific value and too much non-combustible gas must be reasonably controlled; otherwise, a very low or a very high *Φ* is not conducive to the combustion of biogas. There is a quadratic function relationship between the combustion flame propagation velocity and *Φ*.

$$\mathrm{Vf=-77}\mathrm{.76071+264}\mathrm{.9106}\times \Phi \mathrm{-138}\mathrm{.09881}\times \Phi^{2}$$ (3)

Where Φ is equivalence ratio.

**FIGURE 8:**
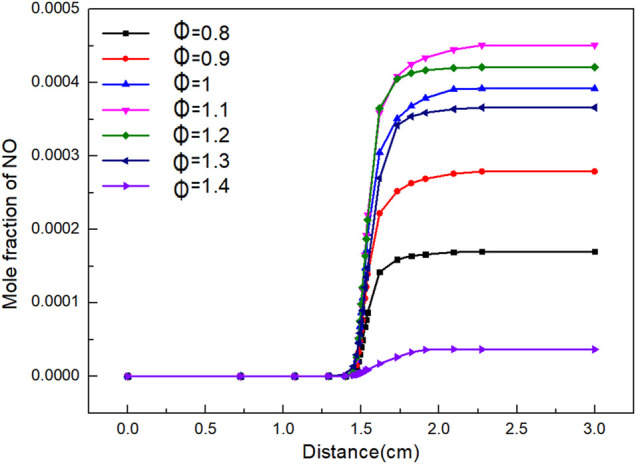
Curve of NO mole fraction with distance under different *Φ*.

From Figure 8, it can be seen that the amount of NO formation increases with the increase of flame distance. When flame distance is 0–1.4 cm, the mole fraction of NO basically remains stable. This is because the combustion temperature of the flame is less than 500 K. When flame distance is 1.4–1.8 cm, the combustion increases sharply due to the rapid transfer of mass and heat. The mole fraction of NO increases greatly when *Φ* is 1.1 and 1.2. When flame distance is 1.4–2.3 cm, the mole fraction of NO increases slowly and finally stabilizes.

**FIGURE 9:**
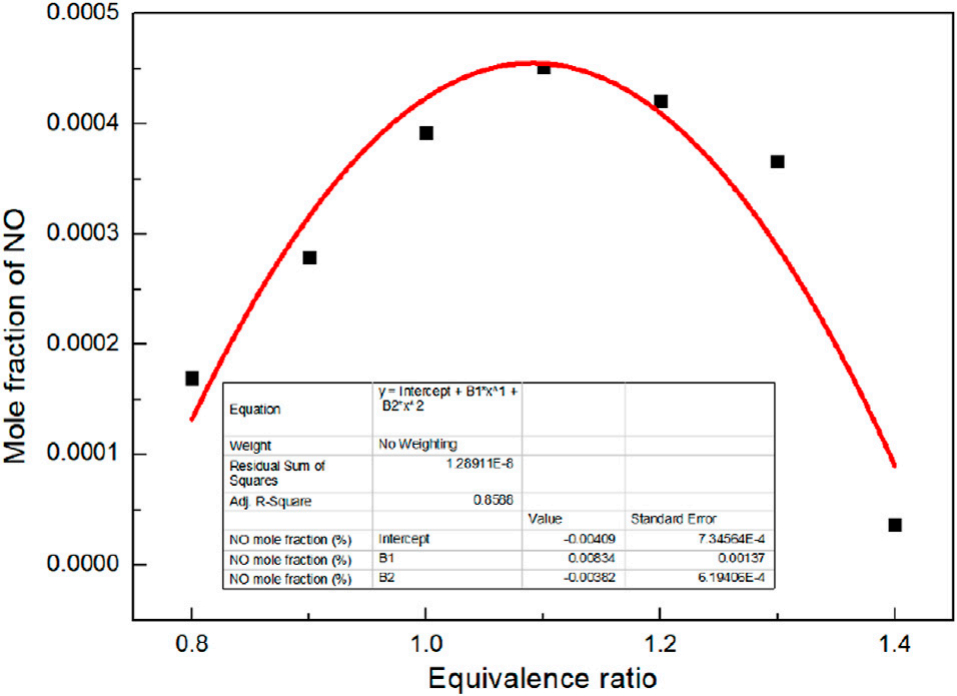
Curve of NO mole fraction with *Φ*.

It can be seen from Figure 9 that the final mole fraction of NO increases firstly and then decreases with the increase of *Φ*. The maximum value of NO appears when *Φ* is 1.1. When *Φ* is 0.8, the biogas cannot be completely burned due to the lack of air, resulting in reducing atmosphere, which can inhibit the formation of nitrogen oxides well. When *Φ* is 1.4, excessive air is introduced to reduce the flame temperature. However, the concentration of nitrogen oxides increases to a certain extent. There is a quadratic function relationship between the NO mole fraction and *Φ*.

$$n\left(\mathrm{NO}\right)=\mathrm{-0}\mathrm{.00409+0}\mathrm{.00634}\times \Phi \mathrm{-0}\mathrm{.00382}\times \Phi^{2}$$ (4)

**FIGURE 10:**
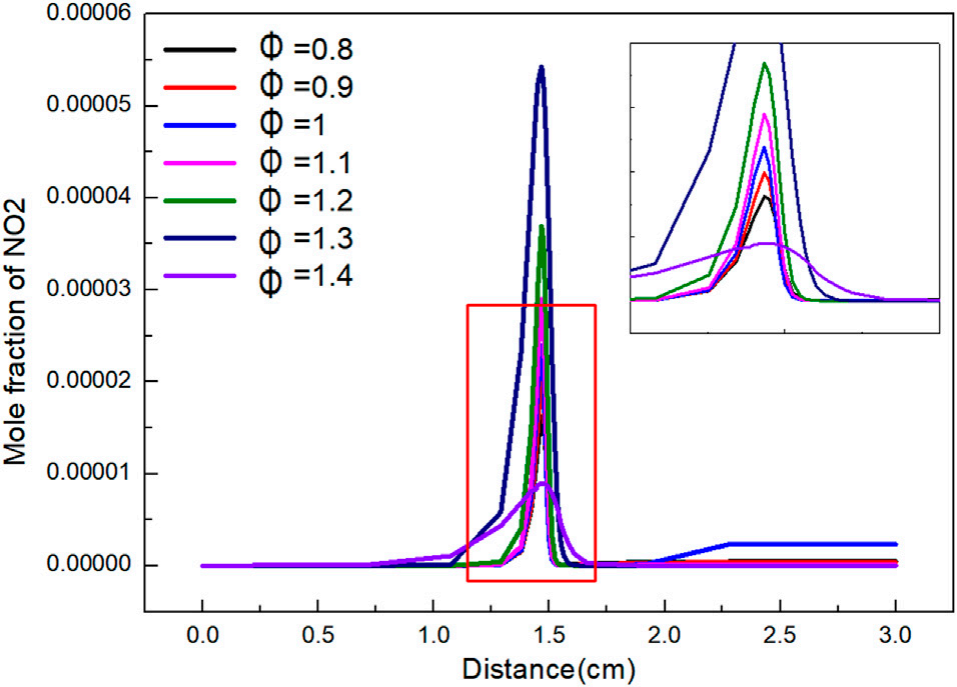
The variation of NO_2_ mole fraction with distance under different *Φ*.

The variation of NO_2_ mole fraction with distance under different *Φ* is shown in Figure 10. The variation trend of NO_2_ mole fraction with distance is roughly the same. When *Φ* is 0.8–1.4, the mole fraction increases firstly, and then reaches the peak at 1.3. The peak value is 5.43 E-07. The lowest peak value was 8.96 E-08 at 1.4. Due to the continuous combustion, the produced NO_2_ is quickly consumed and reaches equilibrium when flame distance is 1.8 cm. The mole ratio of NO_2_ in the final product is much higher than that in other cases when *Φ* is 1. While *Φ* is high, the influence of *Φ* on the proportion of NO_2_ in the final product is not obvious.

### Sensitivity Analysis on Biogas Combustion Temperature

**FIGURE 11:**
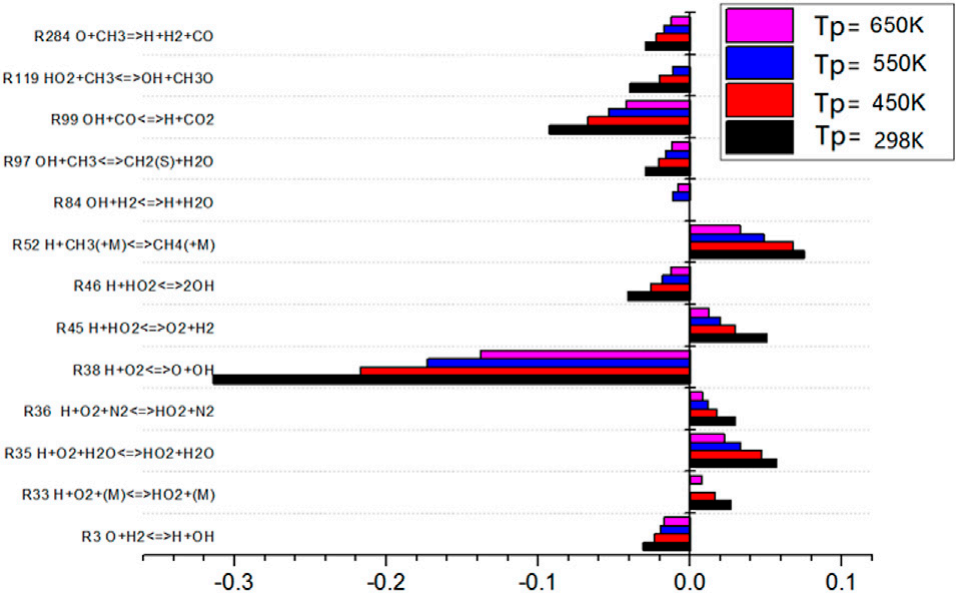
Sensitivity of preheating temperature to combustion temperature.

The sensitivity analysis diagram of the gas preheating temperature to the combustion temperature is as shown in Figure 11. When the preheating temperature changes, the elementary reactions show roughly the same rule. The sensitivity of the elementary reactions decreases with the change of the preheating temperature. It can be seen that the most influential elementary reactions on flame combustion temperature are R38, R99, and R52. Among them, R52 is exothermic reaction, while R38 and R99 are endothermic reactions and absorb heat. With the increase of gas preheating temperature, the heat released by the elementary reaction with positive sensitivity decreases. However, the heat absorbed by the elementary reaction with negative sensitivity decreases more than that with positive sensitivity. The overall effect is that the combustion temperature increases with the gas preheating temperature.

$$\mathrm{R38}\;H+O_{2}<=>\mathrm{O+OH}$$ (5)

$$\mathrm{R52}\;\mathrm{H+C}H_{3}+\left(M\right)<=>CH_{4}+\left(M\right)$$ (6)

$$\mathrm{R99}\;\mathrm{OH+CO<=>H+C}O_{2}$$ (7)

**FIGURE 12:**
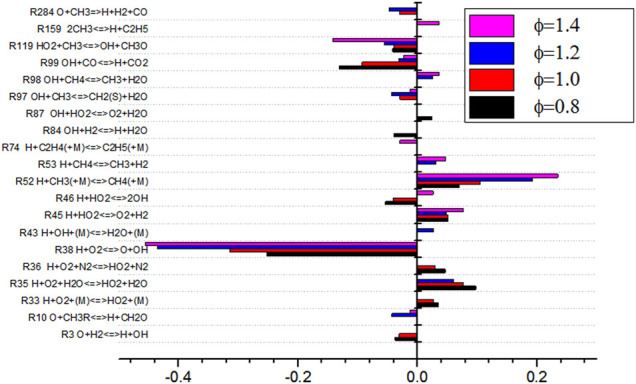
Sensitivity analysis of equivalence ratio to combustion temperature.

As shown in Figure 12, the sensitivity analysis diagram of *Φ* to combustion temperature shows that R38 and R52 (M represents a kind of parameter particle, and the state of it before and after reaction remains unchanged) have the greatest influence on combustion temperature. With the increase of *Φ*, the sensitivity of the reactions of the two elements increases. The sensitivity of R38 is negative and it increases with the increase of *Φ*. The sensitivity of R52 is positive. More heat is released with the increase of *Φ* for R52. The sensitivity of R119 is the highest. Although the sensitivity of R52 is positive, the sensitivity of R35 is negative. The total effect is that the combustion temperature of the flame is the minimum when *Φ* is 1.4.

$$\mathrm{R38}\;H+O_{2}<=>\mathrm{O+OH}$$ (8)

$$\mathrm{R52}\;\mathrm{H+C}H_{3}+\left(M\right)<=>CH_{4}+\left(M\right)$$ (9)

$$\mathrm{R119}\;H0_{2}+CH_{3}<=>\mathrm{OH+C}H_{3}O$$ (10)

**FIGURE 13:**
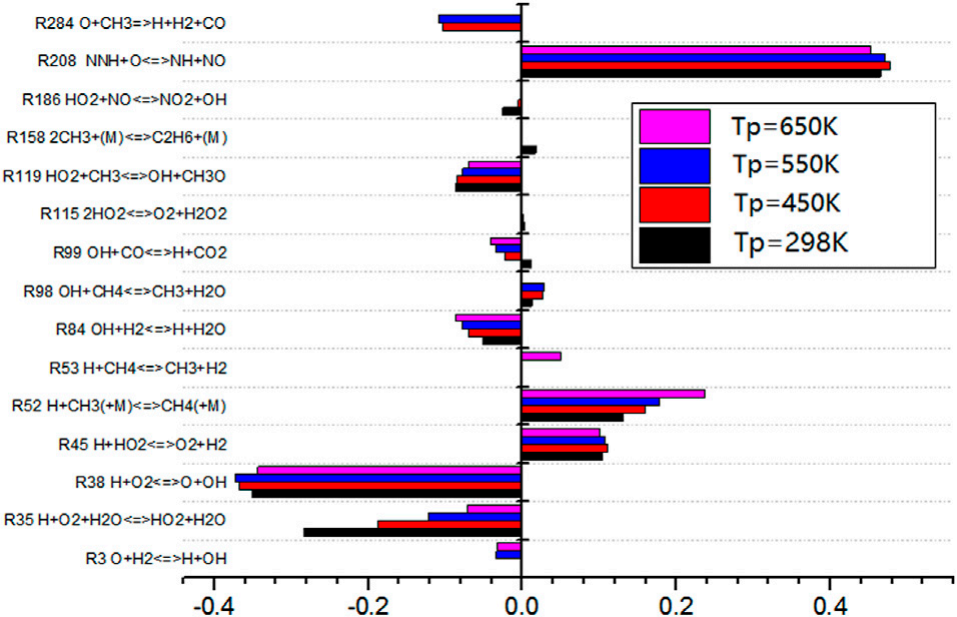
Sensitivity analysis of preheating temperature to NO generation.

As shown in Figure 13, the sensitivity analysis diagram of gas preheating temperature to NO production shows that the sensitivity of elementary reaction R208 to NO is positive under different gas preheating conditions. The value of R208 is the largest, which indicates that this elementary reaction promotes NO production reaction. The promotion effect of R208 is the largest. However, the sensitivity of R38 to NO is negative, which indicates that R38 inhibits NO production and the inhibition effect was the most obvious. With the increase of preheating temperature, the sensitivity of elementary reactions R208 and R38 do not change, but the sensitivity of elementary reactions R35 and R52 changes regularly. With the increase of preheating temperature, the inhibition effect of R35 on NO formation becomes weaker and weaker. The promotion effects of R52 on NO formation become stronger. The higher the preheating temperature, the higher the NO production is.

The sensitivity of R38 to elemental reaction is almost not affected by preheating temperature. However, R38 involves the reaction with oxygen. Therefore, in the combustion reaction of typical low calorific value biogas, it is considered that only by adding an appropriate amount of oxygen will the progress of elemental reaction R38 proceed and will it be more sensitive to NO formation. In this way, the inhibition effect on NO formation occurs. Finally, it can reduce the production of NO.

$$\mathrm{R35}\;H+O_{2}+H_{2}\mathrm{O<=>H}O_{2}+H_{2}O$$ (11)

$$\mathrm{R38}\;H+O_{2}\mathrm{<=>O+OH}$$ (12)

$$\mathrm{R52}\;\mathrm{H+H}O_{2}\mathrm{<=>}O_{2}+H_{2}$$ (13)

R208 NNH+O<=>NH+NO (14)

**FIGURE 14:**
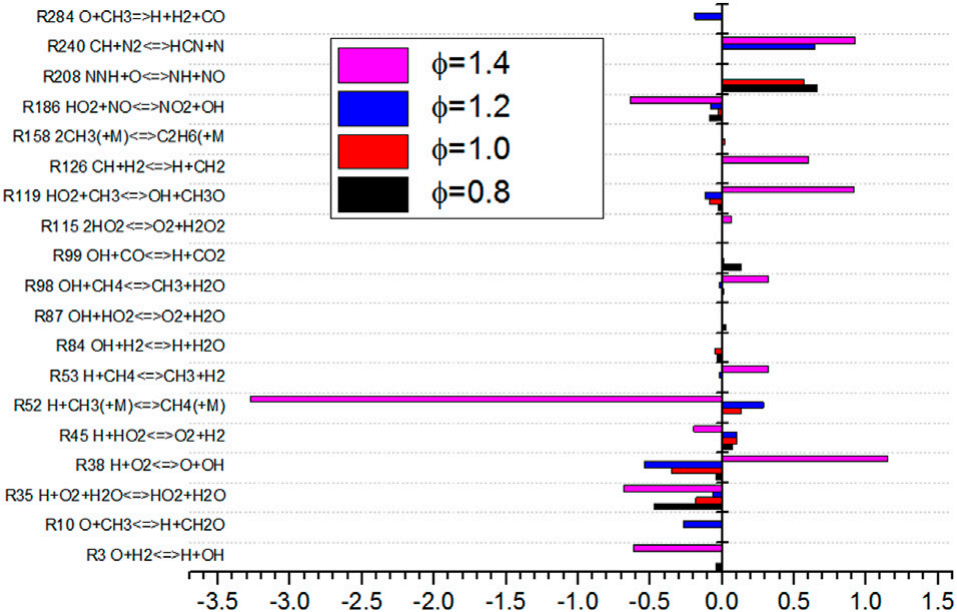
Diagram of sensitivity analysis for NO generation in equivalence ratio.

As shown in Figure 14, the sensitivity analysis diagram of NO production by *Φ* shows that the sensitivity of elementary reaction changes greatly under different equivalence ratios. When the equivalence ratio is lower than 1.4, the sensitivity of the 12 elementary reactions is small, which indicates that there are more elementary reactions that affect NO production process. The absolute value of sensitivity is relatively balanced. When *Φ* is 1.4, it can be seen from Figure 14 that the absolute value of sensitivity of basic reaction R52 is large, which is the most influential basic reaction for NO production. It can also be seen from the figure that under different equivalence ratio conditions, the elementary reactions of R35, R38, R45, R119, and R186 have an impact on the sensitivity of NO production. These reactions participate in the production and consumption of NO. When *Φ* changes from 1.2 to 1.4, the sensitivity of elementary reactions of R38 and R119 changes from negative to positive, while the sensitivity of elementary reactions of R45 and R52 changes from positive to negative. The absolute sensitivity of R52 to NO is much higher than that of other elementary reactions. R52 inhibits the formation of NO. The change of sensitivity of R52 affects the minimum production of NO.

$$\mathrm{R35}\;H+O_{2}+H_{2}\mathrm{O<=>H}O_{2}+H_{2}O$$ (15)

$$\mathrm{R38}\;H+O_{2}\mathrm{<=>O+OH}$$ (16)

$$\mathrm{R45}\;\mathrm{H+H}O_{2}\mathrm{<=>}O_{2}+H_{2}$$ (17)

$$\mathrm{R52}\;\mathrm{H+C}H_{3}+\left(M\right)\mathrm{<=>C}H_{4}+\left(M\right)$$ (18)

$$\mathrm{R119}\;HO_{2}\mathrm{+C}H_{3}\mathrm{<=>OH+C}H_{3}O$$ (19)

$$\mathrm{R186}\;HO_{2}\mathrm{+NO<=>N}O_{2}\mathrm{+NO}$$ (20)

### Reaction Path Analysis

**FIGURE 15:**
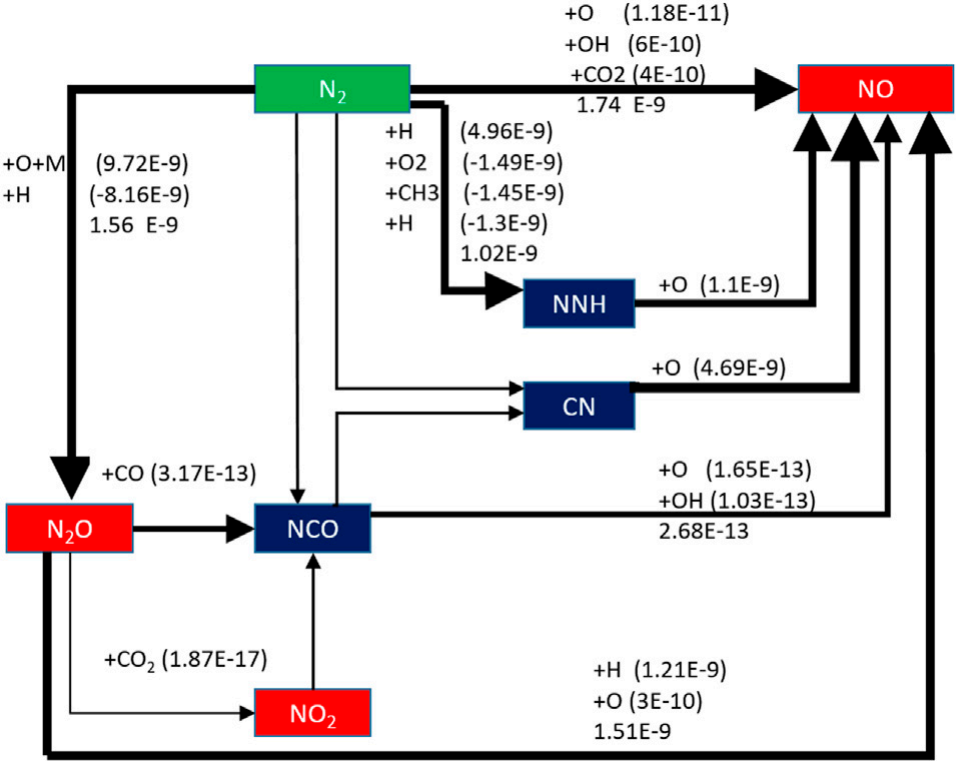
NO generation path after preheat treatment.

The effect of preheating temperature on NO formation path is analyzed when the typical low calorific value biogas is preheated at 650 K. As shown in Figure 15, the NO generation path of biogas becomes more complex when the biomass gas is preheated. The reaction rate of the newly added reaction path is very small compared with the three main paths. Thus, it is not analyzed here.

The three main NO generation paths have improved to varying degrees after the biogas is preheated. The order of magnitude of the reaction rate of the path N_2_ → N_2_O →NO is increased from the original E-10 to E-9. The order of magnitude of the reaction rate of the path N_2_ → NNH →NO is increased from the original E-10 to E-9. The order of magnitude of the reaction rate of the path N_2_ →NO is increased from E-11 to E-9.

It can be seen that the NO formation rate is improved by preheating the biogas. The combustion temperature of the flame will increase accordingly due to the preheating, while the thermal NO formation process is dependent on temperature. So, the reaction rate of N_2_ → NO increases significantly and it becomes the largest of the three reaction paths.

**FIGURE 16:**
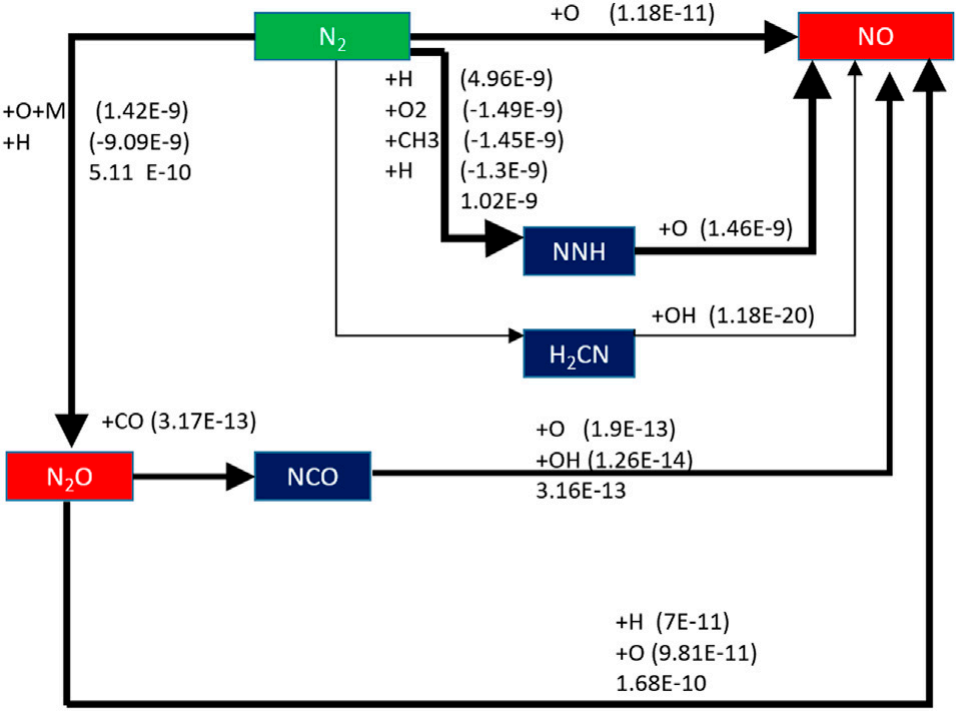
Generation path of NO when equivalent ratio is 0.8.

When the equivalence ratio is changed, the change of NO generation pathway is analyzed. As shown in Figure 16, it can be seen that when *Φ* is 0.8, there is an additional NO formation path: N_2_ → H_2_CN → NO, but the order of reaction rate of this path is only E-20, which has little effect on NO formation.

When equivalence ratio *Φ* is 0.8, the three main reaction paths did not change, but the NO formation rate decreases in varying degrees. The reaction rate of N_2_ → N_2_O →NO only slightly decreased, while the order of magnitude of reaction rate of N_2_ → NNH →NO decreases from E-10 to E-11, and the reaction rate of N_2_ →NO decreased from 5.14 E-11 mol/(cm^3^·s) to 1.18 E-11 mol/(cm^3^·s).

When the equivalence ratio changes from 1.0 to 0.8, the NO formation rates of the three reaction paths decrease; in particular, the reaction rate of N_2_ → NNH →NO decreases obviously. However, the reaction rate of this path is still the highest.

## Conclusion

The combustion characteristics of low calorific value biogas with the variation of preheating temperature and equivalence ratio were discussed. Based on the sensitivity analysis on biogas combustion temperature, the reaction path of NOx was obtained.

1) With the increase of preheating temperature, the final flame temperature increases. When the preheating temperature is 650 K, flame propagation velocity is 75.7 cm/s, about 5 times that without preheating. Preheating of biogas can not only improve the flame propagation speed and flame temperature, but also increase the proportion of NO in the product at the end of combustion flame.

There is a quadratic function relationship between the preheating temperature and NO mole fraction.

$$n\left(\mathrm{NO}\right)\mathrm{\%=2}\mathrm{.93128E-4+2}\mathrm{.89742}\;\mathrm{E-7\times Tp+2}\mathrm{.13244}\;\mathrm{E-9\times T}p^{2}$$ (21)

2) When *Φ* is 1.0, the final flame temperature can reach the highest with 1,675 K, and the final flame temperature is 1,430 K. The maximum value of NO appears when *Φ* is 1.1. There is a quadratic function relationship between the NO mole fraction and *Φ*.

$$n\left(\mathrm{NO}\right)\mathrm{=-0}\mathrm{.00409+0}\mathrm{.00634\times \Phi -0}\mathrm{.00382\times}\Phi^{2}$$ (22)

By analyzing the simulation results, the optimal preheating temperature and equivalence ratio are obtained, which provides theoretical support for industrial boilers to improve combustion efficiency and reduce NOx emissions.

3) The elementary reactions of R35, R38, R45, R119, and R186 have an impact on the sensitivity of NO production. When the equivalence ratio is lower than 1.4, the sensitivity of the 12 elementary reactions is small, which indicates that there are more elementary reactions that affect the NO production process.
4) NO formation rate is improved by preheating the biogas. The paths of N_2_ → N_2_O →NO, N_2_ → NNH →NO, and N_2_ →NO are all enhanced. When the equivalence ratio changes from 1.0 to 0.8, the NO formation rates of the three reaction paths decrease, especially for the reaction rate of N_2_ → NNH →NO.

Through the simulation of the reaction path, the formation path of NO_X_ under different operating parameters has been obtained. Therefore, the generation of NO_X_ can be controlled from the source, which is beneficial to reducing the NO_X_ emission level of industrial burners such as biomass boilers and meets the national emission standards.

In addition, the optimal operation parameters can be obtained by numerical simulation, which reduces unnecessary energy consumption during the operation of industrial boilers.

## Data Availability

The original contributions presented in the study are included in the article/Supplementary Material, further inquiries can be directed to the corresponding author.

## Footnotes

1
http://www.me.berkeley.edu/gri_mech/G.
